# Stereotactic Body Radiation Therapy for Spine Metastases—Findings from an Australian Population-Based Study

**DOI:** 10.3390/curroncol30080564

**Published:** 2023-08-21

**Authors:** Wee Loon Ong, Roger L. Milne, Farshad Foroudi, Jeremy L. Millar

**Affiliations:** 1 Alfred Health Radiation Oncology, Melbourne 3004, Australia; 2 Central Clinical School, Monash University, Melbourne 3004, Australia; 3 School of Clinical Medicine, University of Cambridge, Cambridge CB2 0SP, UK; 4 Cancer Epidemiology Division, Cancer Council Victoria, Melbourne 3004, Australia; 5 Centre for Epidemiology and Biostatistics, School of Population and Global Health, University of Melbourne, Melbourne 3053, Australia; 6 Precision Medicine, School of Clinical Sciences, Monash Health, Monash University, Melbourne 3168, Australia; 7 Department of Radiation Oncology, Olivia Newton-John Cancer Wellness and Research Centre, Austin Health, Heidelberg 3084, Australia

**Keywords:** stereotactic radiotherapy, spine metastases, health services, health disparities

## Abstract

**Background:** To evaluate the use of stereotactic body radiation therapy (SBRT) for spine metastases and the associated factors in Australia. **Methods:** The Victorian Radiotherapy Minimum Dataset, which captures all episodes of radiotherapy delivered in the state of Victoria, was accessed to evaluate the patterns and trends of SBRT for spine metastases. The primary outcome was SBRT use and associated factors. **Results:** There were 6244 patients who received 8861 courses of radiotherapy for spine metastases between 2012 and 2017. Of these, 277 (3%) courses were SBRT, which increased from 0.4% in 2012 to 5% in 2017 (P-trend < 0.001). There was a higher proportion of SBRT use in patients with prostate cancer (6%) and melanoma (4%) compared to other cancers (2–3%) (*p* < 0.001). Patients from the highest socioeconomic quintiles (5%) were more likely to be treated with SBRT compared to patients from the lowest socioeconomic quintiles (3%) (*p* < 0.001). There was a higher proportion of SBRT use in private radiotherapy centres (6%) compared to public radiotherapy centres (1%) (*p* < 0.001). No spine SBRT was delivered in regional centres. In multivariate analyses, the year of treatment, age, primary cancers and radiotherapy centres were independently associated with SBRT use. **Conclusion**: This is the first Australian population-based study quantifying the increasing use of spine SBRT; however, the overall use of spine SBRT remains low. We anticipate an ongoing increase in spine SBRT, as spine SBRT gradually becomes the standard-of-care treatment for painful spine metastases.

## 1. Introduction

Spine metastases are common [1] and can cause severe pain, spinal instability and neurological deficits. Conventional fractionated external beam radiation therapy (cEBRT) is the main management option for painful spine metastases. In patients with metastatic epidural spinal cord compression, open surgical decompression with or without fusion has been the mainstay for preserving and improving neurological function [2]. Over the years, there have been rapid advancements in both surgical techniques (e.g., minimal invasive surgery) and radiotherapy technologies (e.g., stereotactic body radiation therapy (SBRT)) [3]. This has resulted in the management of spine metastases becoming increasingly complex and challenging, requiring multidisciplinary approaches [4], such as the need for separation surgery prior to SBRT [5].

There were three randomised trials [6,7,8] that compared SBRT and cEBRT for painful spine metastases with mixed results [9]. The phase two trial by Sprave et al. showed a trend towards an improved complete pain response but was not powered a priori for superiority [6], while in the RTOG0631 trial, the primary endpoint of superiority of SBRT for pain response at 3 months was not met [8]. To date, the SC24/TROG1706 trial provided the most convincing level one evidence that SBRT for painful spine metastases should be the standard of care [7]. While pain response was the primary endpoint in these three trials, there were other reasons that may have driven an overwhelmingly increased interest in the use of SBRT for spine metastases in recent years [10,11,12]. Firstly, there has been a gradual shift in the philosophy of the oncological management of oligometastatic disease, favouring a metastasis-directed treatment approach with SBRT [13,14]. Secondly, certain cancer histologies are deemed to be more radioresistant and may require a much higher radiation dose to achieve local control [15,16], and SBRT allows for the delivery of a high ablative radiation dose with an extremely steep dose drop-off, sparing the surrounding normal tissues such as the spinal cord. Thirdly, with advancements in novel systemic therapies, patients with spine metastases are living longer and there are predicted to be an increasing number of patients who may experience local failure from previous cEBRT for spine metastases [17] and may benefit from spine reirradiation with SBRT [18].

Our clinical experiences suggest increasing SBRT use in practice; however, there are extremely limited published Australian series on spine SBRT [19,20,21], and the extent of the adoption and actual patterns of SBRT use for spine metastases in Australia is unknown. The aim of this study was to evaluate the patterns of SBRT use for the management of spine metastases in Australia.

## 2. Materials and Methods

### 2.1. Study Sample

This study included a population-based cohort of cancer patients who received radiation therapy for spine metastases between 2012 and 2017, as captured in the Victorian Radiotherapy Minimum Dataset (VRMDS). The VRMDS is a statewide administrative dataset managed by the Victorian Department of Health, which captures all episodes of radiation therapy delivered in all public and private facilities in the state of Victoria in Australia. The purpose of data collection in the VRMDS is to provide relevant data to inform statewide services planning considerations for radiation therapy facilities and the assessment of metrics, such as the radiation therapy utilisation rate [22]. Prespecified data in the VRMDS (such as the number of radiation therapy fractions, target sites of radiation therapy, treatment techniques, etc.) were collected via mandatory self-reporting by each radiation therapy facility, commonly through automated data capture from radiation oncology information systems (e.g., MOSAIQ^®^) or billing data from respective facilities. For this study, we limited the data to radiation therapy to the spine (target site = 24, i.e., *‘radiation therapy directed at the bones of the spine and/or sacrum’*). Patients with primary bone malignancies (ICD-10 code C40-C41) were excluded from the study. The study was approved by our institutional human research ethics committee (LNR/18/34).

### 2.2. Primary Outcomes

The primary outcome of the study was SBRT delivery (yes/no binary outcomes) for spine metastases based on data captured in the VRMDS (treatment technique = 18, i.e., *‘SBRT’*). There were no details on the radiation dose captured in the VRMDS for the study period. Factors potentially associated with SBRT use that were evaluated included: patients’ age at treatment, sex, primary cancer types (confirmed through linkage with the Victorian Cancer Registry), socioeconomic status (derived from residential postcodes using the Socioeconomic Indexes For Areas (SIEFA) index for relative socioeconomic disadvantages and divided into quintiles based on the Victorian general population), area of residence (major cities vs. regional) and the type (public vs. private) and location (metropolitan vs. regional) of the radiation therapy centres.

### 2.3. Statistical Analyses

Categorical variables were described using frequencies and percentages, and differences in characteristics of patients who had SBRT vs. non-SBRT were evaluated using Pearson’s chi-squared test. The Cochran–Armitage test for trends was used to evaluate the temporal trend in SBRT use. Logistic regression was used to evaluate factors associated with SBRT use, and factors with *p* < 0.1 in the univariable analyses were included in the multivariable analyses. Given that a patient could receive multiple courses of radiation therapy at different timepoints over the study period, the robust standard errors were estimated and clustered using patient identifiers to account for multiple courses of radiation therapy in the same patients. A 2-sided *p* < 0.05 was considered to indicate statistical significance. All statistical analyses were performed using Stata/SE 17 (StataCorp, College Station, TX, USA).

## 3. Results

A total of 6244 patients received 8861 courses of radiation therapy for spine metastases. Of these, 277 (3%) courses of radiation therapy were treated with SBRT techniques (Table 1). Different SBRT fractionations were used—58 (21%) were 1 fraction, 11 (4%) 2 fractions, 70 (25%) 3 fractions, 33 (12%) 5 fractions, 86 (31%) 6–10 fractions and 19 (7%) >10 fractions (Table 2).

SBRT was more commonly delivered in younger patients—4% of radiation therapy courses in those aged 60–69 years compared to 2% of those aged ≥ 80 years (*p* < 0.001) (Table 2). Men (4%) were more likely to be treated with SBRT compared to women (2%) (*p* < 0.001). SBRT was more commonly used for spine metastases in primary prostate cancer (6%) and melanoma (4%) compared to lung cancer (2%) (*p* < 0.001). Patients from the highest socioeconomic quintile were more likely to have SBRT (5%) compared to patients from the remaining four socioeconomic quintiles (*p* < 0.001). The proportion of spine radiation therapy that was SBRT was higher in private centres (6%) compared to public centres (1%) (*p* < 0.00). All SBRT was delivered in metropolitan centres and none in regional centres. There was no significant difference in SBRT use for patients who lived in major cities or regional areas (*p* = 0.06).

**Figure 1:**
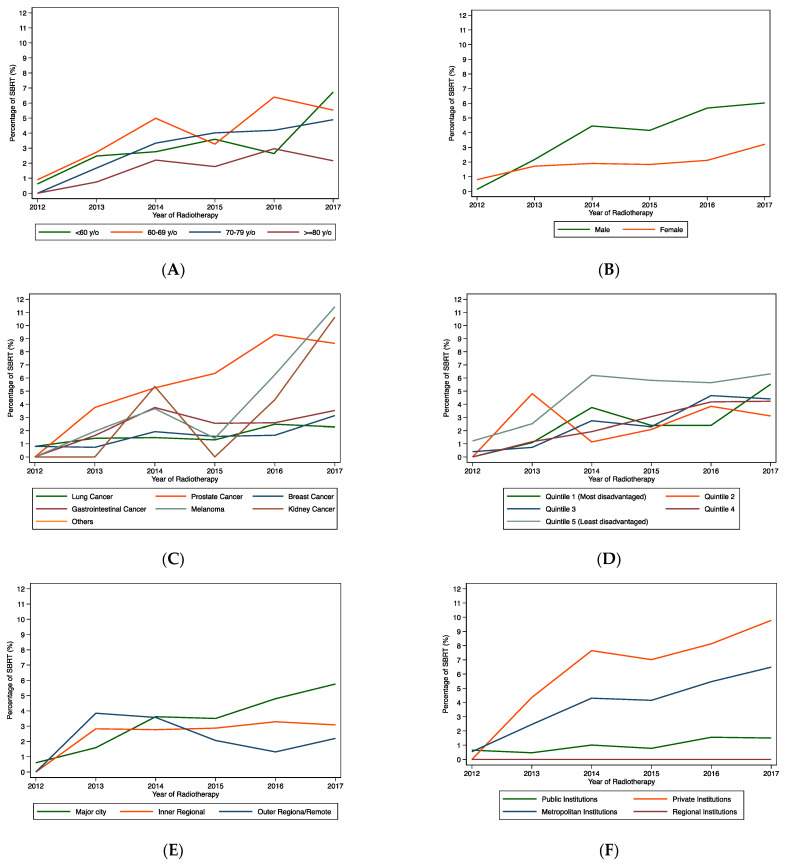
Use of stereotactic body radiation therapy (SBRT) for spine metastases over time, stratified by age (**A**), sex (**B**), primary cancer (**C**), socioeconomic status (**D**), area of residence (**E**) and treatment institution type and location (**F**).

There was a marked increase in SBRT use over time from 0.4% in 2012 to 5% in 2017 (P-trend < 0.001). This progressive increase in SBRT use was observed across the strata of all covariables considered (Figure 1A–F). When stratified by age group, the most marked increase in SBRT use was observed in patients under the age of 60 years—increasing from 0.9% in 2012 to 5.5% in 2017 (P-trend < 0.001) (Figure 1A). There was an increase in SBRT use in both men (from 0.1 in 2012 to 6.0% in 2017) and women (from 0.8% in 2012 to 3.2% in 2017) (Figure 1B). When stratified by primary cancer type, the most marked increase in SBRT use was observed in prostate cancer (from 0% in 2012 to 8.7% in 2017), melanoma (from 0% in 2012 to 11.4% in 2017) and kidney cancer (from 0% in 2012 to 10.6% in 2017) (Figure 1C). A marked increase in SBRT use was also observed in private centres (from 0% in 2012 to 9.8% in 2017) (Figure 1F).

In the multivariable analyses, the increasing use of SBRT over time remained significant after adjusting for other covariables—patients treated in 2017 were an estimated 13 times (95%CI = 5–34; *p* < 0.001) more likely to be treated with SBRT for spine metastases compared to those treated in 2013 (Table 3). Other factors independently associated with SBRT use were age, primary cancer types and type of radiation therapy centres (public/private). Patients aged 80 years and above were significantly less likely to be treated with SBRT (OR = 0.21, 95%CI = 0.11–0.39; *p* < 0.001) compared to those aged under 60 years. Compared to patients with primary lung cancer, those with prostate cancers and melanoma were an estimated 3.9 times (95%CI = 2.3–6.8; *p* < 0.001) and 2.9 times (95%CI = 1.3–6.7; *p* = 0.01) more likely to be treated with SBRT, respectively. Patients treated in private centres were estimated to be 7.4 times (95%CI = 5.3–10.5; *p* < 0.001) more likely to be treated with SBRT compared to patients treated in public centres.

## 4. Discussion

This was the first Australian population-based study evaluating the pattern of SBRT use for spine metastases. We observed an increasing use of SBRT for spine metastases over time; however, SBRT use remained low at only 5% of all radiation therapy for spine metastases in 2017. Age, primary cancer type and treatment centre type were factors independently associated with SBRT use, and no spine SBRT was delivered in regional centres.

There are very few similar population-based studies in the literature reporting on the pattern of spine SBRT use over time in other countries. The only other population-based study evaluating the pattern of spine SBRT was based on US data from the National Cancer Database (NCDB) (Table 4) [23,24]. Of the 89,025 patients who had radiation therapy for spine metastases between 2004 and 2013 in the US study, 1030 (1.2%) were treated with SBRT, and this increased from 1.4% in 2004 to 5.8% in 2013. Overall, there appeared to be an earlier adoption of SBRT use for spine metastases in the US compared to Australia. Despite these two studies covering slightly different periods, when we looked at the overlapping period between our study and that reported in the NCDB (i.e., 2012 and 2013), the proportion of SBRT use for spine metastases in our cohort (0.4–2.0%) was lower than that reported in the US (5.7–5.8%) [23].

Nonetheless, since then, there have been several tumour-specific SBRT clinical trials in Australia that may have led to increasing SBRT use for spine metastases over our study period (2012–2017)—this included the POPSTAR trial (2013–2014) for prostate cancer [25] and the BOSTON trial (2014–2016) for breast cancer [26], of which approximately half of the patients had spine metastases. There was also the SC24/TROG1706 trial (2016–2019), which randomized patients with spine metastases to SBRT vs. cEBRT [7]. It is of interest to note that while the POPSTAR and BOSTON trials both used 20 Gy in one-fraction SBRT [25,26] and SC24/TROG1706 trial used 24 Gy in two-fraction SBRT [7], only 25% of SBRT in the current study employed one–two-fraction SBRT (Table 2). However, it was also important to recognize that there has been no proven ‘standard of care’ dose fractionation for spine SBRT until the publication of the results of the SC24/TROG1706 trial in 2021; hence, outside of recruiting centres putting patients in trials, heterogeneity in practice and the choice of dose fractionation would be expected over our study period.

In the current study, we identified several factors associated with SBRT use for spine metastases. Younger patients were more likely to be treated with SBRT in our study, which was consistent with findings from the US [24]. This was most likely attributable to the selection of younger patients who were estimated to have a sufficiently long survival to benefit from higher and more prolonged local control from SBRT compared to cEBRT [27]. Increasing age is generally considered a negative prognostic factor in most cancers and is included in multiple predictive models as a factor associated with poorer survival after palliative radiation therapy for cancers [28,29]. SBRT also requires rigid treatment immobilisation and longer on-table treatment time, and may cause more stress, therefore, is not as well-tolerated by elderly patients.

Another factor that influences survival in cancer patients with spine metastases is the primary cancer type. We observed that patients with prostate cancer and melanoma were more likely to be treated with SBRT, and these are cancers that are often associated with better prognoses with multiple novel systemic therapy options [30,31,32]. The higher SBRT use for spine metastases in prostate cancer may also have been driven by the recruitment of men into phase two of the POPSTAR trial over the study period [25], and the subsequent experience and comfort in offering SBRT for spine metastases in prostate cancer. It is of interest to note that in the US study, using the NCDB database, McClelland et al. observed highest SBRT use for spine metastases in patients with primary lung cancer [23]. However, the authors acknowledged that this may have been due to limitations of the use of the NCDB database, such that they may have underestimated the true number of patients with prostate cancer who had radiation therapy for spine metastases [23]. Another possible reason for the difference in the use of SBRT by primary cancer type was that certain cancers, such as melanoma and renal cell carcinoma, are generally considered to be radioresistant and may need a higher radiation dose to improve local control [15].

We also observed institutional variations in SBRT use, whereby patients treated in private centres were more likely to be treated with SBRT, while no SBRT was delivered in regional centres. In the study in the US, it was reported that patients with private insurance, those who lived in metropolitan areas and those who had treatment in academic centres were more likely to be treated with SBRT [24] (Table 4, suggesting possible disparities in access to SBRT for spine metastases. There were various possible reasons for the observed institutional differences in SBRT use in our study. It is important to acknowledge that SBRT can be resource-intensive. There are various personnel and equipment requirements for the establishment of a comprehensive SBRT program for the safe delivery of spine SBRT [33]. Private and public facilities may differ in drivers and constraints in developing SBRT programs and using SBRT in different clinical situations. One also could not discount the possibility that the institutional differences in SBRT use may be remuneration-related. In the current Australian healthcare setting, reimbursement is based on the number of fractions and radiation therapy techniques—the reimbursement for single-fraction spine SBRT is AUD 4208.25, while the reimbursement for single-fraction cEBRT is AUD 1320.35–1948.80 (depending on the number of fields and the complexity of the cEBRT involved) [34]. In addition, as part of spine SBRT, it is critical that the spinal cord is accurately contoured in order to reduce the risk of radiation myelopathy [35]. This requires either image fusion with magnetic resonance imaging or CT myelogram [36], which may not be easily accessible in all centres, especially in regional centres. Reassuringly, patients who live in regional or remote areas were equally likely to be treated with SBRT in our study (Table 2), suggesting that they may have been referred on to metropolitan centres for SBRT when clinically indicated.

A major strength of this study using the administrative VRMDS was that it captured all episodes of radiation therapy delivered in all radiation therapy facilities in the state of Victoria and, hence, reflected a true statewide pattern of practice. However, there were several limitations of the study, which were inherent limitations of the VRDMS. The VRMDS is dependent on coding and reporting from individual radiation therapy facilities to the Department of Health, and we could not discount the possibilities of miscoding or data entry errors, which may have led to the misclassification of primary outcomes and covariables. In particular, SBRT courses of >5 fractions may either have been errors in the coding of the radiation therapy techniques as ‘SBRT’ or errors in the reporting of the number of fractions delivered. However, it was not possible for us to verify this based on the available data in the VRMDS. From 2018 onwards, information on radiation dose was captured in the VRMDS, and the availability of information on the dose-per-fraction allowed us to crosscheck if the radiation therapy was true ‘SBRT’, especially in cases of >5-fraction SBRT. There was also a lack of granularity in the VRMDS for us to evaluate the appropriateness of the SBRT use in each individual patient. For example, there was no clinical information to ascertain if a treated spine metastasis was associated with spinal instability [37], pathological fracture or epidural spinal cord compression [38]. There was no information about surgical interventions prior to the radiation therapy, and we were not able to evaluate if there were differences between SBRT and cEBRT use in postoperative settings. There was no information about the level of spine irradiated and we were not able to ascertain if patients who had multiple courses of radiation therapy had reirradiations of the same level of spine. Given that the VRMDS only captures information on radiation therapy, there was also a lack of data on the combination of SBRT with systemic therapies such as immunotherapy [39]. There was also a lack of important spine SBRT outcomes data in the dataset, including local control and symptom control outcomes, as well as toxicity outcomes such as vertebral fracture [40,41] and radiation myelopathy [35].

Moving forward, as spine SBRT gradually becomes a standard-of-care treatment for spine metastases [9], there is a need to ensure that the current healthcare system is able to cope with the increasing demand for spine SBRT in the coming years [42]. While we observed an overall increasing trend in the use of SBRT for spine metastases in the current study, only approximately 5% of spine metastases were treated with SBRT in 2017, and there were no spine SBRT services available in regional centres. It is well-recognized that there is often a time lag in the adoption of new radiation therapy techniques and technologies into routine clinical practice [43], and there can also be variation in access to, and the receipt of, these novel technologies among cancer patients, as has been observed with the use of stereotactic radiosurgery (SRS) for brain metastases [44,45,46,47]. To meet the expected increasing demand for spine SBRT, it is important that appropriate funding and resources are put into establishing and developing frameworks for the expansion of dedicated spine SBRT programs across multiple radiation oncology departments in Victoria, including in regional centres, for the safe delivery of spine SBRT. These include the training of clinicians through mentorship programs (similar to the UK SABR Consortium) to ensure consistency in practice across all centres with respect to contouring, as well as interpretation and the adherence of consensus recommendations and guidelines [48,49]. There is also a requirement for safe radiation therapy simulations, image guidance and quality assurance programs, such as those recommended by the Canadian Association of Radiation Oncology SBRT Task Force [50]. At the same time, it is also important to further strengthen the multidisciplinary care coordination between spine surgeons and radiation oncologists, as the concept of separation surgery is also becoming increasingly common prior to spine SBRT [5,51,52]. In situations where there are spinal metastases with epidural extension, separation surgery is required to create sufficient spatial distance between the tumour and spinal cord to ensure the safe delivery of a high SBRT dose to the tumour without causing damage to the spinal cord.

## 5. Conclusions

In conclusion, this population-based study showed an increase in SBRT use for spine metastases from 2012 to 2017 in Australia. However, as of 2017, only 5% of spine metastases were treated with SBRT, and no spine SBRT was delivered in regional centres. It would be of great interest to evaluate the pattern of spine SBRT practice in more recent years. As cancer patients with spine metastases are living longer, the management of spine metastases is likely to become increasingly complex and challenging, requiring close collaboration between spine surgeons and radiation oncologists. While SBRT is gradually becoming the standard-of-care in the management of painful spine metastases, it is crucial that the current healthcare system is well-prepared for the increasing demand for spine SBRT. At the same time, in our effort to reduce disparities and increase access to spine SBRT for all cancer patients across all sociodemographic groups in whom spine SBRT is clinically indicated, it is equally important to ensure that robust spine SBRT programs are well-established to ensure the safe delivery of spine SBRT.

## Figures and Tables

**Table 1:** Baseline characteristics of spine metastases treated with radiation therapy.

	Non-SBRT	SBRT	*p*-Value
	8584 (97%)	277 (3%)	
Age at radiation therapy			
Mean (SD)	68.4 (13.3)	67.2 (10.9)	0.1
<60	2043 (97%)	67 (3%)	<0.001
60–69	2337 (96%)	99 (4%)	
70–79	2455 (97%)	80 (3%)	
≥80	1749 (98%)	31 (2%)	
Sex			
Male	5078 (96%)	207 (4%)	<0.001
Female	3506 (98%)	70 (2%)	
Primary cancer			
Lung	1853 (98%)	31 (2%)	<0.001
Prostate	2066 (94%)	129 (6%)	
Breast	1740 (98%)	30 (2%)	
Gastrointestinal	1001 (98%)	25 (2%)	
Melanoma	323 (96%)	12 (4%)	
Renal cell carcinoma	358 (97%)	11 (3%)	
Others	1243 (97%)	39 (3%)	
Socioeconomic status			
First quintile (lowest)	1721 (97%)	47 (3%)	<0.001
Second quintile	1375 (98%)	35 (2%)	
Third quintile	1549 (97%)	40 (3%)	
Fourth quintile	1786 (97%)	49 (3%)	
Fifth quintile (highest)	2153 (95%)	106 (5%)	
Remoteness of residence			
Major cities	5978 (97%)	211 (3%)	0.06
Inner regional	2139 (97%)	56 (3%)	
Outer regional/remote/very remote	467 (98%)	10 (2%)	
Type of radiation therapy centres			
Public	5350 (99%)	54 (1%)	<0.001
Private	3234 (94%)	223 (6%)	
Location of radiation therapy centres			
Metropolitan	6629 (96%)	277 (4%)	<0.001
Regional	1955 (100%)	0 (0%)	
Year of radiation therapy			
2012	1212 (99.6%)	5 (0.4%)	<0.001
2013	1383 (98%)	28 (2%)	
2014	1498 (97%)	53 (3%)	
2015	1658 (97%)	56 (3%)	
2016	1449 (96%)	64 (4%)	
2017	1384 (95%)	71 (5%)	

SBRT—stereotactic body radiation therapy.

**Table 2:** Number of fractions for all courses of stereotactic body radiation therapy (SBRT) (*n* = 277).

Number of Fractions	Number of SBRT Courses
1 fraction	58 (21%)
2 fractions	11 (4%)
3 fractions	70 (25%)
5 fractions	33 (12%)
6–10 fractions	86 (31%)
>10 fractions	19 (7%)

**Table 3:** Multivariate analyses of factors associated with stereotactic body radiation therapy (SBRT) for spine metastases.

	Adjusted OR (95%CI)	*p*-Value
Age at radiation therapy		
<60	Reference	
60–69	0.84 (0.55–1.29)	0.4
70–79	0.48 (0.31–0.75)	0.001
≥80	0.21 (0.11–0.39)	<0.001
Sex		
Male	Reference	
Female	0.82 (0.51–1.31)	0.4
Primary cancer		
Lung	Reference	
Prostate	3.91 (2.26–6.77)	<0.001
Breast	0.86 (0.44–1.70)	0.7
Gastrointestinal	1.23 (0.65–2.34)	0.5
Melanoma	2.90 (1.25–6.70)	0.01
Renal cell carcinoma	1.75 (0.76–3.99)	0.2
Others	2.06 (1.14–3.74)	0.02
Socioeconomic status		
First quintile (lowest)	Reference	
Second quintile	0.74 (0.42–1.32)	0.3
Third quintile	0.82 (0.48–1.42)	0.5
Fourth quintile	0.65 (0.39–1.08)	0.1
Fifth quintile (highest)	1.53 (0.97–2.41)	0.07
Remoteness of residence		
Major cities	Reference	
Inner regional	0.79 (0.52–1.20)	0.3
Outer regional/remote/very remote	0.66 (0.31–1.40)	0.3
Type of radiation therapy centres		
Public	Reference	
Private	7.41 (5.24–10.47)	<0.001
Year of radiation therapy		
2012	Reference	
2013	4.79 (1.72–13.3)	0.003
2014	9.25 (3.53–24.26)	<0.001
2015	8.84 (3.41–22.94)	<0.001
2016	11.55 (4.45–29.99)	<0.001
2017	13.01 (5.05–33.52)	<0.001

**Table 4:** Population-based studies reporting on stereotactic body radiation therapy (SBRT) use for spine metastases.

	Data Source	Study Period	N	SBRT Use	Factors Associated with SBRT Use
McClelland, 2017 [23] Kim, 2021 [24]	National Cancer Database (NCDB), USA	2004–2013	89,025 *	Overall: 1030 (1.2%) 2004: 1.4% 2013: 5.8%	Age, race, insurance status, area of residence, comorbidities and treatment centres
Current study	Victorian Radiotherapy Minimum Dataset (VRMDS), Victoria, Australia	2013–2017	8584 ^#^	Overall: 277 (3%) 2012: 0.4% 2017: 5.0%	Age, primary cancer and treatment centres

* Number of patients; ^#^ number of courses of radiation therapy.

## Data Availability

Research data are stored in an institutional repository and can be shared upon reasonable request to the corresponding author.
